# Pseudoperoxidase activity, conformational stability, and aggregation propensity of the His98Tyr myoglobin variant: implications for the onset of myoglobinopathy

**DOI:** 10.1111/febs.16235

**Published:** 2021-11-03

**Authors:** Stefan Hofbauer, Marcello Pignataro, Marco Borsari, Carlo Augusto Bortolotti, Giulia Di Rocco, Gianina Ravenscroft, Paul G. Furtmüller, Christian Obinger, Marco Sola, Gianantonio Battistuzzi

**Affiliations:** ^1^ Department of Chemistry Institute of Biochemistry University of Natural Resources and Life Sciences Vienna Austria; ^2^ Department of Chemical and Geological Sciences University of Modena and Reggio Emilia Italy; ^3^ Department of Life Sciences University of Modena and Reggio Emilia Italy; ^4^ Harry Perkins Institute of Medical Research Nedlands WA Australia; ^5^ School of Biomedical Sciences University of Western Australia Nedlands WA Australia

**Keywords:** conformational stability, heme bleaching, high‐molecular‐weight aggregates, myoglobin, myoglobinopathy

## Abstract

The autosomal dominant striated muscle disease myoglobinopathy is due to the single point mutation His98Tyr in human myoglobin (MB), the heme protein responsible for binding, storage, and controlled release of O_2_ in striated muscle. In order to understand the molecular basis of this disease, a comprehensive biochemical and biophysical study on wt MB and the variant H98Y has been performed. Although only small differences exist between the active site architectures of the two proteins, the mutant (a) exhibits an increased reactivity toward hydrogen peroxide, (b) exhibits a higher tendency to form high‐molecular‐weight aggregates, and (c) is more prone to heme bleaching, possibly as a consequence of the observed H_2_O_2_‐induced formation of the Tyr98 radical close to the metal center. These effects add to the impaired oxygen binding capacity and faster heme dissociation of the H98Y variant compared with wt MB. As the above effects result from bond formation/cleavage events occurring at the distal and proximal heme sites, it appears that the molecular determinants of the disease are localized there. These findings set the basis for clarifying the onset of the cascade of chemical events that are responsible for the pathological symptoms of myoglobinopathy.

## Graphical abstract

The mutation His98Tyr in human myoglobin induces an autosomal dominant muscle disease (myoglobinopathy). The mutant exhibits (a) an increased reactivity towards H_2_O_2_ as well as (b) a higher tendency to form high‐molecular‐weight aggregates, and (c) is more prone to heme bleaching, possibly due to the observed H_2_O_2_‐induced formation of a Tyr98 radical. These findings set the bases for clarifying the onset of the chemical events responsible for the pathological symptoms of myoglobinopathy.

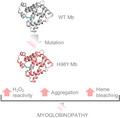

**Abbreviations**
EGFP: enhanced green fluorescent protein
GSH: glutathione
HEK cells: human embryonic kidney cells
MALS: multi‐angle static light scattering
MB: human myoglobin
PDA: photodiode‐array detection
ROS: reactive oxygen species
SEC: size‐exclusion chromatography
swMB: sperm whale myoglobin

## Introduction

Myoglobin (MB) and its partner in O_2_ transport, hemoglobin, have been studied intensively for decades [1]. MB is a single‐chain 14 kDa molecular weight (MW) protein with a heme group (iron protoporphyrin IX) wedged into a hydrophobic pocket featuring a 5‐coordinate iron atom [1]. The sixth iron coordination position can be occupied by ligands such as O_2_, CO, and NO [1]. Beyond the two major roles—storage and facilitation of O_2_ diffusion in the heart and skeletal muscle—recent studies revealed that MB may protect the cell against reactive oxygen species (ROS) [2] and regulates NO homeostasis, but can also have detrimental effects depending on tissue oxygen partial pressure and ROS availability [1, 3, 4, 5]. This variety of roles is related to the rich redox chemistry of MB as it yields multiple protein forms depending on the oxidative conditions of the cell [4]. These include oxygen‐bound form (MBFe(II)O_2_), deoxymyoglobin (MBFe(II)), metmyoglobin (MBFe(III)), cross‐linked MB, and the ferryl forms MBFe(IV)=O and MB^•+^(IV)=O [4]. In addition, dissociation of ferriprotoporphyrin IX from MB and release of iron atoms may occur, contributing to various oxidative pathologies in muscles [6].

**Fig. 1:**
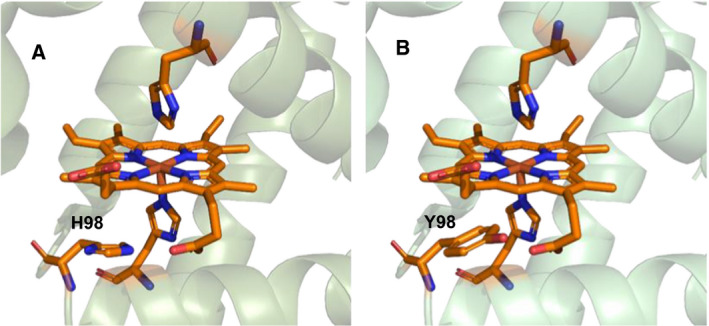
Active site of human myoglobin (pdb code: 3rgk); (A) wt MB, (B) model of H98Y MB variant; figure was constructed using PyMOL.

Recently, a recurrent His98Tyr substitution in human myoglobin (MB) (Fig. 1A,B) was identified as the determinant of myoglobinopathy, a rare autosomal dominant myopathy, featuring highly characteristic sarcoplasmic inclusions in skeletal and cardiac muscle [7]. The disease manifests in adulthood with proximal and axial muscle weakness and progresses to cause wheel‐chair dependence and respiratory and cardiac failure [7]. H98Y MB shows a decreased O_2_ binding affinity, a larger heme dissociation rate, and a lower reduction potential (*E*°') compared with wt MB [7]. The comprehension of the molecular basis of the disease requires a much deeper biochemical characterization of the variant. In particular, additional work is required to bring to light the differences between the H98Y and wt MB in the interplay between the above‐mentioned redox forms and the interaction with relevant metabolites under a variety of conditions. This would open the way to the understanding of the molecular mechanism(s) leading to the disease and its progressive evolution, and to the development of strategies to slow down or inhibit disease progression. In this work, we tackled this complex issue pursuing a twofold objective, namely to elucidate how the H98Y substitution affects the electronic structure of the heme center and to assess the reactivity of mutant MB toward hydrogen peroxide. His97 in sperm whale myoglobin (*sw*MB)—corresponding to H98 in human MB—is an important surface amino acid hydrogen bonded to heme propionate‐7 (p7 hereafter) which, along with other residues of the proximal side of the heme pocket, plays a critical role in the retention of the heme group [8, 9]. Substitutions at His97 in *sw*MB involving disruption of the H‐bond with p7 were found to induce structural changes at the heme propionates and the strengthening of the axial Fe(III)‐*ε*N(His93) bond, along with the decrease in iron displacement from the heme plane [8]. Therefore, the H98Y mutation in human MB could result in similar structural changes at the heme site worth investigating. Hydrogen peroxide is a typical ROS that also functions as secondary messenger in the cell [10, 11, 12]. The reaction between MB and H_2_O_2_ has been thoroughly investigated [3, 13, 14, 15, 16]. It is well known that MB possesses a peroxidase‐like activity [3, 17] that relies on the formation of a ferryl group, and therefore may participate in antioxidant defense by detoxifying endogenous H_2_O_2_ by reducing substrates (ascorbate, GSH, vitamin E) [3]. On the contrary, ferryl‐MBs were shown to induce lipid peroxidation [18]. Moreover, H_2_O_2_ promotes formation of cross‐linked MB [14, 15, 19, 20, 21], is responsible for lipid oxidation in low‐density lipoproteins and phospholipids [22] and heme–protein cross‐link [20, 23], and can also lead to heme degradation (heme bleaching) [4, 19]. The released ferriprotoporphyrin IX is a strong oxidant that promotes lipid oxidation [4].

Since human embryonic kidney cells (HEK cells) transfected with H98Y MB‐EGFP featured intracellular superoxide levels higher (1.45‐fold) than those transfected with wild‐type MB‐EGFP [7], the interaction of H98Y MB with H_2_O_2_ may in principle favor reaction cascades responsible for the onset of myoglobinopathy. These reactions require the involvement of protein radicals, such as cysteine thiyl and tyrosyl radical [21, 24, 25, 26, 27]. In this respect, the insertion of a new solvent‐exposed tyrosine in close proximity of the heme in H98Y MB is very important. In fact, providing another site for tyrosyl radical formation, it may alter reaction pathways leading to potentially harmful heme–protein and protein–protein cross‐linkages. Here, we found that the studied variant, although possessing a conserved active site compared with wt MB, shows indeed an altered reactivity with H_2_O_2_. Larger binding rates and increased pseudoperoxidase activity (detoxifying action) were observed on the one hand and larger H_2_O_2_‐induced tendency to form high‐MW aggregates and to undergo heme bleaching (harmful effects) on the other. The balance between these effects most likely plays a role in the onset of the pathological symptoms of myoglobinopathy.

## Results and Discussion

### Electronic structure of the heme site

The EPR spectra of wt and H98Y variant of MBFe(III) (Fig. S1A) show minor differences in the peak shape of the Fe(III) signal. In particular, the wt protein yields a completely axial signal with a very sharp peak, whereas the variant shows a slightly broader peak, but still completely axial. The simulated spectra are shown in Fig. S1A. Simulation parameters for wt MB are as follows: g_1_ = g_2_ = 5.92, g_3_ = 1.995; g_strain1_ = g_strain2_ = 0.15, g_strain3_ = 0.1; rhombicity = 0%. The parameters for the H98Y variant are as follows: g_1_ = g_2_ = 5.913, g_3_ = 1.995; g_strain1_ = g_strain2_ = 0.2, g_strain3_ = 0.14; rhombicity = 0%. These simulations show very minor differences of the electron distribution of the heme iron, which is mainly reflected in the peak width. The g_1_/g_2_ region indicates that the active site architecture in solution is slightly different in the H98Y variant. This is the only perceivable spectroscopic difference of the H98Y variant against wt MBFe(III), as the UV‐vis spectra are identical [7].

### Comparing protein conformation and thermal stability: CD spectra and differential scanning calorimetry

The far‐UV CD spectra for wt and H98Y MB (Fig. S1B) are almost identical, indicating that the overall structure is conserved and therefore that the MB structure undergoes only minor rearrangements upon H98Y substitution, in agreement with previous MD simulations and aggregation test [7]. The melting curve at 222 nm (Fig. S2A) reveals that the H98Y variant has a higher melting temperature *T*_m_ than wt MB, indicating that the substitution increases the stability of the protein toward thermal unfolding. This is confirmed by the differential scanning calorimetry measurements (Fig. S2B). Analysis of the melting temperature at different pH values (Fig. S2C) reveals that H98Y is invariably more stable toward thermal denaturation throughout the pH range investigated, although the difference decreases with increasing pH. Both species exhibit maximal stability at pH 6.5. Hence, the H98Y mutation induces myoglobinopathy by affecting MB properties other than those directly related to the overall protein fold, which is conserved [7], and its thermal stability.

### Kinetics of CN^−^ binding

Cyanide binding to wt and H98Y MBFe(III) yields in both cases a six‐coordinate low‐spin CN^−^ derivative, in which the exogenous ligand occupies the distal coordination position of the ferric heme (shown by the Soret band at 424 nm and one band at 540 nm, Fig. S3A). Calculation of the rate constants, *k*_on_, of CN^−^ binding from the slope of the linear plots of *k*_obs_ values versus CN^−^ concentration (*k*_obs_ = *k*_on_[CN^−^] + *k*_off_; Fig. S3B) yielded apparent bimolecular binding rates of 171 and 114 M^−1^·s^−1^ for wt and H98Y MB, respectively (Table 1). From the intercept of the linear plots, the dissociation rate constants (*k*_off_) were obtained allowing calculation of the dissociation constants, *K*_D_ (= *k*_off_/*k*_on_), which resulted 3.7 and 10.9 µm, respectively (Table 1). Ferric H98Y MB therefore features a lower affinity for cyanide compared with the wt protein, as previously observed for molecular oxygen [7]. Hence, it appears that the H98Y substitution causes a decrease in the affinity of heme for exogenous ligands, independently of the Fe oxidation state.

**Table 1:** Kinetics and *K*_D_ of CN^−^ binding to wt MBFe(III) and its H98Y variant at pH 7.4.

	*k*_on_ (M^−1^s^−1^)	*k*_off_ (s^−1^)	*K*_D_ (µm)
wt MB	171 ± 2	(6.3 ± 5) × 10^−4^	3.7
H98Y MB	114 ± 3	(1.2 ± 1) × 10^−3^	10.9

### Kinetics of Compound I formation and heme degradation with hydrogen peroxide

**Fig. 2:**
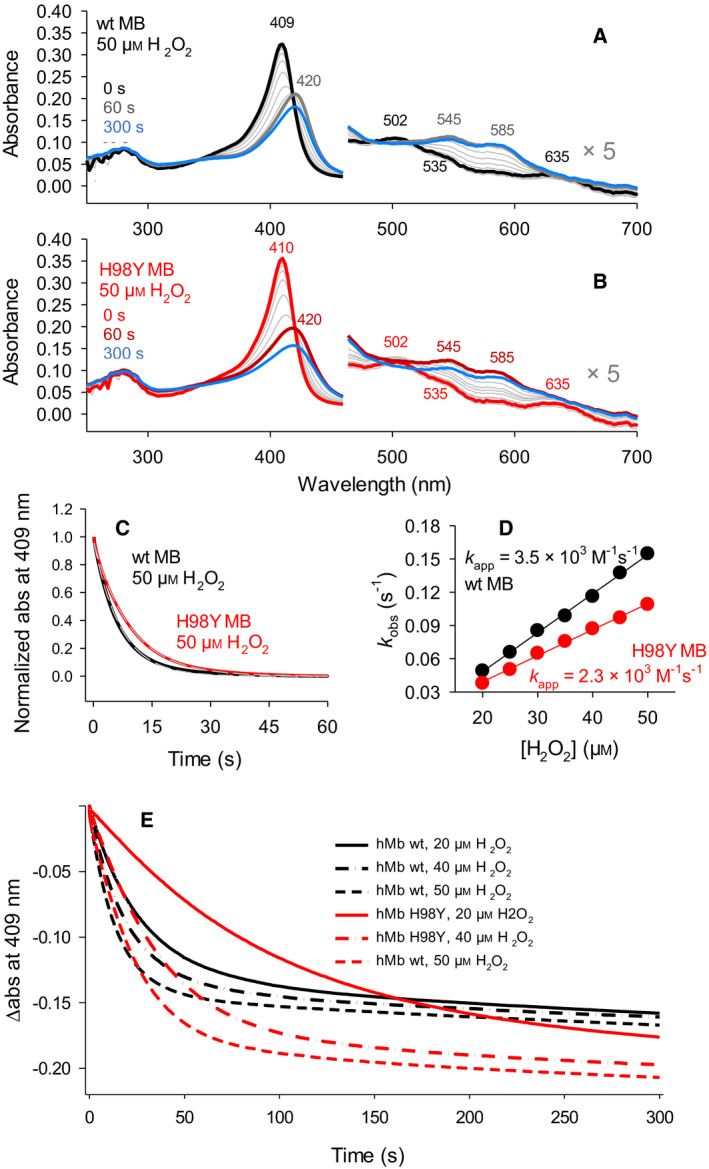
Compound I formation and heme degradation of 3 µm wt MB (black) and H98Y MB (red), followed by stopped‐flow UV‐vis spectroscopy. Spectral changes for (A) 3 µm wt MBFe(III) and (B) 3 µm H98Y MBFe(III) variant occurring in the first 60 s after addition of 50 µm hydrogen peroxide. The effect of heme degradation on longer reaction times is shown by the blue spectra, recorded after 300 s. (C) Time course of the normalized absorbance change at 409 nm fitted single exponentially for both wt MB (black) and H98Y MB (red). (D) *k*_obs_ values for wt MB (black) and H98Y MB (red) plotted against hydrogen peroxide concentration to obtain the apparent rate constants (*k*_app_) for Compound I formation from the slope of the plots. (E) Time course of the absorbance changes at 409 nm occurring during the reaction of wt MB and H98Y MB with hydrogen peroxide for a time span of 300 s. Heme degradation after Compound I formation was manifested in a biphasic loss of absorbance at 409 nm.

The time‐resolved Compound I formation for wt MBFe(III) and the H98Y MBFe(III) variant with hydrogen peroxide was studied by stopped‐flow UV‐vis spectroscopy in the conventional mixing mode. Compound I formation can readily be followed by its reduced absorbance in the Soret region. The transition of wt MB and H98Y MB to Compound I (featuring distinct peaks at 420, 545, and 585 nm; Fig. 2A,B) is overlaid by the decay of the latter, shown by the decrease of its absorption bands (Fig. 2A,B) [13]. This process is generally attributed to an intramolecular ET from Compound I to the sidechain of an amino acid, possibly a tyrosine [28]. The insertion of Y98 close to the heme could explain the increased kinetics of this process in the mutant, leading to an Y98 tyrosyl radical (see below). Consistent with previous literature [13, 19], the reaction of MB and of the H98Y variant on a longer timescale (600 s) shows heme bleaching after ferryl species formation (Fig. 2). From the time course of the spectral changes (Fig. 2), we could determine the kinetic constants for Compound I formation and heme degradation (Fig. 2C–E, Table 2). Compound I formation for H98Y MB variant is 1.5 times slower compared with wt, while the rate of heme degradation is enhanced (Table 2). The kinetic constants of both processes decline with increasing pH for either wt or H98Y MB (Fig. S4A,B). However, the mutation‐induced changes in the kinetics of heme bleaching decrease progressively at high pH and almost vanish at pH 8.7. The larger rate of heme bleaching for H98Y MB is consistent with its higher tendency to lose the heme, indicated by the faster heme release [7], and with the loss of the electrocatalytic properties of the mutant at H_2_O_2_ concentration at which wt MB is still active (see below).

**Table 2:** Kinetic constants for the formation of Compound I and heme degradation for wt MB and its H98Y variant interacting with H_2_O_2_ at pH 7.4.

	Compound I formation *k*_app_ (M^−1^s^−1^)	Heme degradation *k* (s^−1^)
wt MB	(3.5 ± 0.7) × 10^3^	(2.52 ± 0.02) × 10^−3^
H98Y MB	(2.3 ± 0.6) × 10^3^	(4.94 ± 0.01) × 10^−3^

### Formation of a tyrosyl radical at Y98 during H_2_O_2_‐mediated turnover

**Fig. 3:**
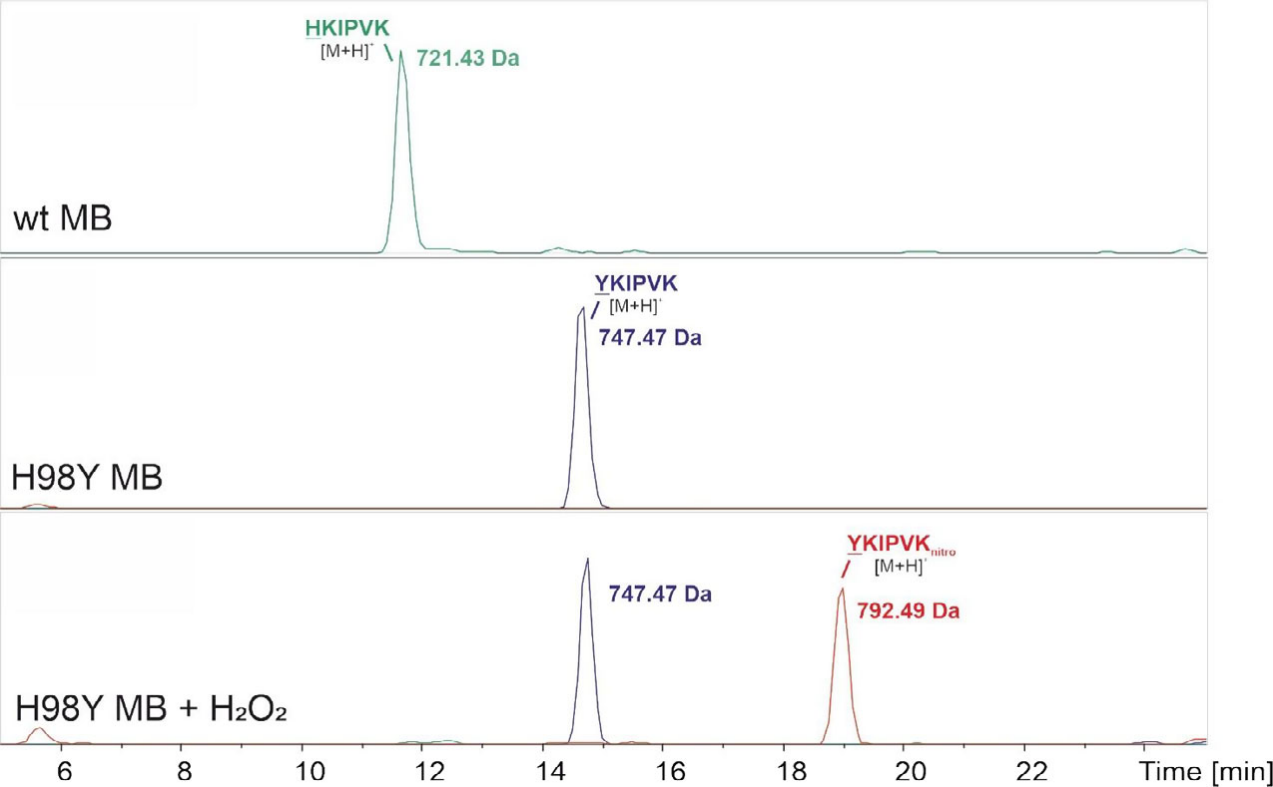
HPLC‐MS analysis of peptides containing the H98 of wt MB and Y98 of the H98Y MB variant. Top panel: LC chromatogram of untreated wt MB showing a single HKIPVK peptide with a mass of 721.43 Da (green); Middle panel: LC chromatogram of untreated H98Y variant showing a single YKIPVK peptide with a mass of 747.47 Da (blue); Bottom panel: LC chromatogram of the H98Y variant treated with hydrogen peroxide and nitrite showing that a significant portion of the YKIPVK peptide is nitrosylated (addition of 45 Da; 792.49 Da) (red).

To prove the existence of the Y98 radical during H_2_O_2_ turnover of H98Y MB, nitrosylation of wt and H98Y MB was tested. Both proteins were treated with excess hydrogen peroxide in the presence of NO_2_^‐^ and subjected to a subsequent HPLC‐mass spectrometry analysis. wt MB shows the peptide 98‐103 (HKIPVK), which features the native His at position 98, at 721.43 Da (Fig. 3, top panel), whereas the corresponding peptide of H98Y MB (YKIPVK) has a mass of 747.47 Da (Fig. 3, middle panel). Upon mixing H98Y MB with NO_2_^‐^ in the presence of hydrogen peroxide, the YKIPVK peptide with a nitrosylated Tyr98 could be detected at 792.49 Da (Fig. 3, bottom panel). These results clearly identify Y98 as radical species during the H_2_O_2_‐mediated turnover reaction. As also native Y104 and Y147 were shown to be nitrosylated and to form radicals during turnover with hydrogen peroxide, we repeated these experiments under the same conditions and confirmed the published results (data not shown) [16].

### H_2_O_2_‐induced protein aggregation

**Fig. 4:**
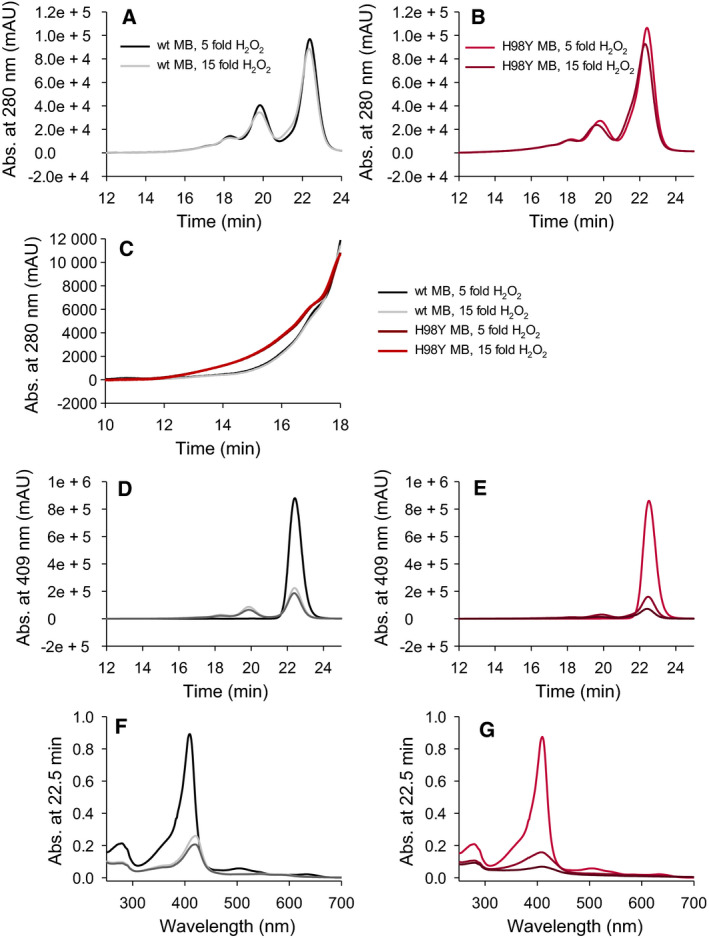
HPLC chromatograms of untreated wt MB (black), wt MB incubated with a fivefold (light gray), and a 15‐fold (dark gray) excess of hydrogen peroxide for 1 h followed at 280 nm (A) and 409 nm (D). HPLC chromatograms of untreated H98Y MB (red) and H98Y MB incubated with a fivefold (dark red) and a 15‐fold (brown) excess of hydrogen peroxide for 1 h at 280 nm (B) and 409 nm (E). Comparison between portions (elution times of 10–18 min) of the HPLC chromatograms at 280 nm of wt MB and H98Y MB incubated with a fivefold (black and dark red, respectively) and a 15‐fold (light gray and red, respectively) excess of hydrogen peroxide for 1 h (C). UV‐vis spectra of the peaks eluted at 22.5 min for wt MB (F) and H98Y MB (G).

WT and H98Y MB were also subjected to HPLC‐SEC‐PDA‐MALS analysis before and after reaction with hydrogen peroxide. For both untreated proteins, only monomers were found, while after 1‐h incubation with a fivefold and 15‐fold excess H_2_O_2_, the chromatograms revealed also the presence of dimers, trimers/tetramers, and other larger aggregates (Fig. 4). Although the overall aggregation patterns of wt MB and the H98Y mutant are quite similar in the chromatograms obtained from the absorbance at 280 nm (Fig. 4A,B), the elution of oligomeric protein starts significantly earlier in the H98Y mutant than in the wt (11.7 min versus approximately 14 min, Fig. 4C). Moreover, the H98Y mutant yields a higher amount of aggregates with a heme/monomer ratio lower than 1, in line with its larger sensitivity to heme bleaching compared to wt MB (Fig. 4). The tendency of H98Y MB to form higher molecular weight aggregates could be related to the presence of the phenolic ring of Tyr98 in close proximity of the heme and the reduced stability of the heme cavity due to the absence of the salt bridge between His98 and the heme propionate. We propose that tyrosyl radical formation (which often occurs at the start of aggregation events in proteins) and the increased heme bleaching of the H98Y mutant following interaction with H_2_O_2_ (and thereby to ROS in general) concur to the formation of protein aggregates with high MW (Fig. 4C). This fits with the abnormal protein aggregation, pronounced release of ferric ions, and high lipid oxidation observed in the sarcoplasmic bodies associated with myoglobinopathy [7]. It therefore would appear that the increased sensitivity to ROS of the H98Y mutant plays a key role in the development of myoglobinopathy. The UV‐vis absorption spectra of all detected LC‐chromatography peaks after reaction with hydrogen peroxide show that the wt protein retains a higher *Reinheitszahl* (RZ) (A_Soret_/A_280_) than the H98Y variant (Fig. 4A,B,D–G). The loss of Soret band absorbance reflects the loss of correctly bound heme and therefore heme bleaching, which is definitely more pronounced in the H98Y variant.

### Electrocatalytic reduction of H_2_O_2_ by wt and H98Y MB measured by cyclic voltammetry

Cyclic voltammograms for MB and H98Y MB were obtained by embedding the proteins into a hydrogel made of type A gelatin and drop casting the construct on a polycrystalline graphite electrode at pH 7.4. Such procedure has been shown to be useful for allowing proteins [29, 30, 31, 32, 33, 34], and particularly globins [35, 36], to talk to electrodes without suffering denaturation.

Typical CVs are shown in Fig. S5A,B (black trace). Both proteins display a signal arising from the quasi‐reversible one‐electron electron transfer process of the heme iron. The *E*°′ values of −0.059 and −0.108 V obtained at pH 7.4 for wt and H98Y MB, respectively, compare well with those obtained for hemoglobin in similar conditions [35, 37] and are lower than the values obtained for the freely diffusing protein [38, 39, 40, 41], confirming a selective stabilization of the ferric globins due to gelatin‐embedding [35].

**Fig. 5:**
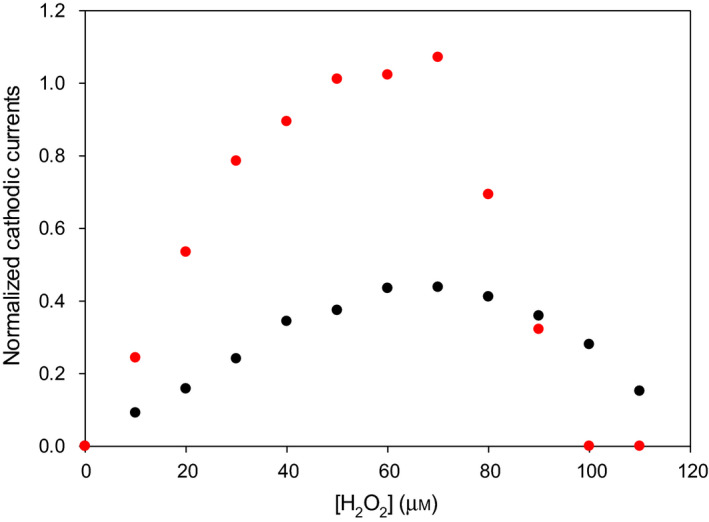
Normalized cathodic currents for the electrocatalytic reduction of H_2_O_2_ by wt MB (black) and H98Y MB (red) adsorbed onto a polycrystalline glassy carbon electrode through embedment into a hydrogel made of type A gelatin at pH 7.4 in the presence of increasing H_2_O_2_ concentrations.

The cyclic voltammograms for electrode‐immobilized MB and H98Y MB, recorded in the presence of increasing micromolar concentrations of H_2_O_2_ at pH 7.4, are shown in Fig. S5. The cathodic currents were found to progressively increase with increasing substrate concentration, with a typical catalytic behavior, indicating that both adsorbed proteins catalyze H_2_O_2_ reduction (‘pseudoperoxidase’ activity). A similar activity was detected previously for electrode‐immobilized wt yeast iso‐1‐cytochrome *c* (ycc) [42] subjected to urea‐induced unfolding [43, 44] or upon cardiolipin binding [45], for its K72A/K73H/K79A mutant [46], as well as for five‐coordinate M80A ycc variants [47, 48, 49]. No additional peaks were detected in the potential range from +0.6 to −0.8 V at all hydrogen peroxide concentrations investigated. The catalytic currents for wt MB increase up to 60 μm H_2_O_2_ and decrease for H_2_O_2_ concentration higher than 70 μm, while H98Y MB yields currents that are up to three times higher and that increase up to 70 μm H_2_O_2_ for eventually decreasing abruptly and vanishing at H_2_O_2_ concentration of about 100 μm (Fig. 5). Elimination of H_2_O_2_ by rinsing the electrode with buffer restored the CV signal of the protein throughout the H_2_O_2_ concentration range investigated for wt MB and only below 60 μm H_2_O_2_ for H98Y MB. The catalytic currents could then be re‐obtained upon H_2_O_2_ addition. Therefore, under these conditions the protein layer is rather stable and reusable.

The catalytic activity of the electrode‐immobilized species can be estimated using the Michaelis–Menten model expressed in terms of current and reported as Lineweaver–Burk plots (Equation 1) [42, 46, 47, 48, 49]:

(1) $$\begin{array}{ll} 1/i_{cat\;normalized}= & 1/i_{max\;normalized}\\ & +K_{M}/\left(i_{max\;normalized}\cdot \left[H_{2}O_{2}\right]\right) \end{array}$$

where *i_cat normalized_* is the normalized electrocatalytic current (*i_cat_/i_0_*), and *i_max normalized_* is the normalized maximum current at substrate saturation (*i_max_/i_0_*). The Lineweaver–Burk plots at pH 7.4 (Fig. S6) yield the *i_max normalized_* and *K*_M_ values listed in Table 3. We propose the following mechanism of hydrogen peroxide electrocatalysis carried out by the five‐coordinate heme of MB at the observed potentials (Equations (2), (3), (4)) [3, 4, 13, 42, 48, 49, 50]:

(2) $$\text{MBFe}\left(\text{III}\right)‐{\text{OH}}_{2}\;+\;e^{‐}\;\to \;\text{MBFe}\left(\text{II}\right)\;+\;H_{2}O$$

(3) $$\begin{array}{ll} \text{MBFe}\left(\text{II}\right)\;+ & \;H_{2}O_{2}\;⇄\;\left[\text{MBFe}\left(\text{II}\right){‐}^{‐}O_{2}H\;+\;H^{+}\right]\\ & \;\to \;\text{MBFe}\left(\text{IV}\right)\;=\;O\;+\;H_{2}O \end{array}$$

(4) $$\text{MBFe}\left(\text{IV}\right)\;=\;O\;+\;e^{‐}\;+\;{\text{2 H}}^{+}\;\to \;\text{MBFe}\left(\text{III}\right)‐{\text{OH}}_{2}$$

**Table 3:** *i_max normalized_* and *K*_M_ values for the electrocatalytic reduction of H_2_O_2_ by wt MB and its H98Y variant at *T* = 293 K and pH 7.4, immobilized on a polycrystalline graphite electrode.

	*i_max normalized_*	*K*_M_ (µm)	*i_max normalized_/K*_M_ (µm ^−1^)
wt MB	1.56 ± 0.06	163.3 ± 6.5	(9.55 ± 0.76) × 10^−3^
H98Y MB	4.53 ± 0.18	169.8 ± 6.8	(2.68 ± 0.21) × 10^−2^

The H98Y variant displays identical *K*_M_ and larger *i_max normalized_* values compared with wt MB (Table 3). This is indicative of a conserved kinetic instability of the primary Fe(II)‐hydroperoxide complex, which is in agreement with the conserved structural and electronic features of the heme center indicated by the present EPR data and MD calculations [7]. The larger catalytic activity of the H98Y variant, indicated by the increased *i_max normalized_*/*K*_M_ ratio (Table 3), shows that the ferryl group in the variant is a more efficient oxidant toward various substrates compared with wt. Moreover, it undergoes autoxidation to a larger extent as demonstrated by the dramatic drop of the catalytic activity for H_2_O_2_ concentrations above 60 μm, which is likely due to uncontrolled oxidative processes involving free radicals leading to protein degradation and to the release of the heme group in solution. Such inactivation occurs for metmyoglobin and methemoglobin [51, 52, 53, 54, 55, 56], several heme peroxidases [57, 58, 59, 60, 61], and other heme proteins [55, 62, 63, 64, 65, 66]. It is conceivable that *in vivo* the larger ‘pseudoperoxidase’ activity of the variant compared with wt MB contributes to the increased ROS production in HEK cells transfected with H98Y Mb‐EGFP [7]. This in turn could be responsible for the formation of the protein aggregates and the unbalancing of the NO production observed in striated muscle of myoglobinopathy patients. Moreover, the inability of the H98Y mutant to mediate the electrocatalytic reduction of H_2_O_2_ at substrate concentrations at which the wt protein is still electroactive confirms its increased sensitivity to heme bleaching.

## Conclusions

The H98Y substitution in human MB is responsible for myoglobinopathy, a progressive myopathy characterized by sarcoplasmic inclusions in skeletal and cardiac muscle. [7] Over the past decades, dozens of myoglobin mutants have been expressed to investigate structure–function relationships and to modify the catalytic properties of the protein, but, to the best of our knowledge, the H98Y substitution has not been investigated. In this work, we have found that the mutant has a 3D structure more stable than wt MB in the acidic‐neutral pH range. On the other hand, upon reaction with hydrogen peroxide, H98Y loses more easily the heme group and shows an increased protein cross‐linking compared with wt. As the mutant also shows a lower affinity for O_2_ and CN^−^, we conclude that an altered interaction of the heme center with the exogenous 6th axial ligand and with the protein environment, along with the presence of a potential tyrosyl radical close to the heme, is the major molecular determinant of the disease onset. These results require validation from *in vivo* studies on effective oxygen level and consumption, as well as on unbalanced ROS production from the free iron.

## Materials and methods

### Materials

Both human wt MB and its H98Y variant were produced and purified as previously reported [7].

### Electron paramagnetic resonance spectroscopy

Spin state determination of ferric wt MB and its H98Y variant was probed by electron paramagnetic resonance (EPR) on a Bruker EMX continuous‐wave (cw) spectrometer, which was operating at X‐band (9 GHz) frequencies. The instrument was equipped with a high sensitivity resonator and an Oxford Instruments ESR900 helium cryostat for low‐temperature measurements. Spectra were recorded under nonsaturating conditions using 2 mW microwave power, 100 kHz modulation frequency, 1 mT modulation amplitude and 40 ms conversion time, 40 ms time constant, and 2048 points. Samples of recombinant human wild‐type myoglobin and the variant H98Y (100 µL of 50 μm) were prepared in 1 × phosphate buffer saline (PBS) buffer, pH 7.4, transferred into Wilmad quartz tubes (3 mm inner diameter), and flash‐frozen in liquid nitrogen. In order to avoid the artifact of a broad paramagnetic signal, caused by O_2_, the tubes were flushed with argon, while the sample was kept frozen on dry ice. Measurements were performed at 10 K. The spectra were simulated with the Easyspin toolbox for MATLAB [67] and consist of a weighted sum of simulations of the individual high‐spin and low‐spin species. The rhombicity was obtained from g_x_^eff^ and g_y_^eff^, and the relative intensities were calculated on the basis of the simulations, following the procedure of Aasa & Vanngard to account for the different integral intensity per unit spin of species that display different effective g‐values (as found in potential low‐spin and high‐spin centers) [68].

### Circular dichroism measurement

Circular dichroism (CD) and absorption spectra were recorded (Chirascan, Applied Photophysics, Leatherhead, UK) between 260 and 200 nm (with 5 s·nm^−1^ scan speed, 1‐nm bandwidth, 1‐mm pathlength, and 5 μm protein concentration). Experiments were conducted in 25 mm phosphate and 25 mm acetate buffer at different pH values. The instrument was flushed with a nitrogen flow of 5 L·min^−1^. UV–vis electronic absorption and circular dichroism spectra were simultaneously recorded. The thermal denaturation was followed at 222 nm, heating from 20 °C to 90 °C with a heat rate of 1 °C·min^−1^. The melting temperature was calculated by fitting the obtained curve with a sigmoid function.

### Differential scanning calorimetry

Differential scanning calorimetric (DSC) measurements were performed using an automated PEAQ‐DSC (Malvern Panalytical) with a cell volume of 130 µL. The measurements were controlled by the integrated Microcal PEAQ‐DSC software, and the instrument was equipped with an autosampler for 96‐well plates. Samples were analyzed using a programmed heating scan rate of 60 °C·h^−1^ over a temperature range from 20 °C to 100 °C, cell pressure was approximately 60 psi (4.136 bar). Thermograms were corrected for buffer baseline and protein concentration. 10 μm of wt, or 10 μm H98Y MB variant in 1 × PBS buffer, pH 7.4, was used for each measurement. For data analysis and conversion, the integrated analysis software was used. Heat capacity (*C*_p_) was expressed in kJ·mol^−1^·K^−1^. Data points were fitted to non‐two‐state equilibrium‐unfolding models by the Levenberg–Marquardt (LM) nonlinear least squares method.

### Stopped‐flow UV‐vis measurements

The stopped‐flow apparatus (model SX 18MV, Applied Photophysics) was equipped with a diode‐array detector and an optical quartz cell with a path length of 10 mm (volume: 20 μL). The fastest time of mixing was 1 ms. All measurements were performed at 25 °C. The protein concentration in the cell was 3 μm. The kinetics of interaction and degradation of the ferric (met) form of wt and H98Y MB with hydrogen peroxide were measured in 25 mm phosphate buffer, pH 7.0, and 25 mm acetate buffer, pH 5.0. Compound I formation was determined by monitoring both the polychromatic light in the visible range and the absorbance decrease at 409 nm. The apparent second‐order rate constants, *k*_app_, were obtained from the slope of a plot of *k*_obs_ values versus the H_2_O_2_ concentration; *k*_obs_ values were obtained by time traces at 409 nm, fitted for 25 s to obtain Compound I formation rate and for 500 s, fitted from 40 s to the end, to obtain heme bleaching. Heme bleaching was measured only at high hydrogen peroxide concentration (larger than 5 mm). Cyanide binding was measured by following the spectral transition from the Soret maximum (409 nm) at the high‐spin state to 424 nm at the low‐spin state. Three determinations were performed for each cyanide concentration. The mean of the pseudo‐first‐order rate constants, *k*_obs_, measured at 409 nm, was used in the calculation of the second‐order rate constant (*k*_on_) obtained from the slope of the plot of *k*_obs_ versus ligand concentration, and *k*_off_ was derived from the intercept with the *y*‐axis of this plot.

### Detection of tyrosyl radicals in wt MB and H98Y MB

In order to detect nitrosylated tyrosine residues of wt MB and H98Y [16], 20 μm of the respective protein was treated with a 10‐fold excess of hydrogen peroxide and 10‐ to 100‐fold excess of NO_2_^−^. 20 µg of each sample in 1×PBS were S‐alkylated with iodoacetamide and further digested with sequencing grade modified trypsin (Promega, Madison, Wisconsin, USA). 5 µg of the peptide mixture was analyzed using a Dionex Ultimate 3000 HPLC system (Thermo Fischer Scientific, Waltham, Massachusetts, USA) directly linked to a QTOF instrument (maXis 4G ETD, Bruker, Billerica, Massachusetts, USA) equipped with the standard ESI source in the positive ion, data‐depended acquisition (DDA) mode (= switching to MSMS mode for eluting peaks). MS scans were recorded (range: 150–2200 m·z^−1^, spectra rate: 1.0 Hz), and the 5 highest peaks were selected for fragmentation. Instrument calibration was performed using ESI tuning mix (Agilent, Santa Clara, California, USA). Peptides were separated using a Thermo BioBasic C18 separation column (5 µm particle size, 150 × 0.320 mm). A gradient from 96% solvent A and 4% solvent B (Solvent A: 65 mm ammonium formate buffer, pH 3.0; Solvent B: 80% ACN and 20% A) to 40% B in 35 min was applied, followed by a 15‐min gradient from 40% B to 94% B, at a flow rate of 6 µL·min^−1^ at 32 °C. Manual glycopeptide searches were done using DataAnalysis 4.0 (Bruker, Billerica, Massachusetts, USA).

### HPLC‐SEC‐PDA‐MALS measurements

Analyses were performed on an LC20 prominence HPLC system equipped with the refractive index detector RID‐10A, the photodiode‐array detector SPD‐M20A (all from Shimadzu, Japan), and a MALS Heleos Dawn8+ plus QELS detector (Wyatt Technology, Santa Clara, California, USA). The column (Superdex 200 10/300 GL, GE Healthcare, Chicago, Illinois, USA) was equilibrated with 1 × PBS plus 200 mm NaCl (pH 7.4) as running buffer. Experiments were performed at a flow rate of 0.75 mL·min^−1^ at 25 °C and analyzed using the astra 6 software (Wyatt Technology, Santa Clara, California, USA). Proper performance of molar mass calculation by MALS was verified by the determination of a bovine serum albumin sample. All proteins were incubated for 1 h at room temperature with zerofold, fivefold and 15‐fold excess (calculated as molar ratio H_2_O_2_/MB) of H_2_O_2_ before being centrifuged (at 15 000 ***g*** for 2 min at 20 °C), filtered (0.1‐mm Ultrafree‐MC filter, Merck Millipore, Darmstad, Germany), and finally analyzed. A total amount of 60 µg was injected for all measurements.

### Electrochemical measurements

A potentiostat/galvanostat mod. 273A (EG&G PAR, Oak Ridge, TN, USA) was used to perform cyclic voltammetry (CV). Experiments were carried out using a cell for small volume samples (0.5 mL) under argon. A 1‐mm‐diameter graphite disk electrode, a Pt wire, and a saturated calomel electrode (SCE) were used as working, counter, and reference electrode, respectively. The electrical contact between the SCE and the working solution was achieved with a Vycor® (from PAR) set. Reduction potentials were calibrated against the MV^2+^/MV^+^ (MV = methyl viologen) and ferrocene/ferrocenium couples under all experimental conditions employed in this work to make sure that the effects of liquid junction potentials were negligible. All the reduction potentials reported here are referred to the standard hydrogen electrode (SHE), unless otherwise specified. The working electrode was cleaned before each use with mechanical polishing with 0.5 μm aluminum oxide followed by 5‐min immersion in an ultrasonic bath. The Vycor® set was treated in an ultrasonic pool for about 5 min. Protein solutions were freshly prepared before use in 20 mm phosphate buffer at pH 7.4, and their concentration (typically 100 μm) was carefully checked spectrophotometrically (Jasco mod. V‐570 spectrophotometer). Wt MB and H98Y MB were immobilized through embedment into a hydrogel made of type A gelatin and adsorbing the construct on a polycrystalline graphite electrode at pH 7.4 [29, 30, 31, 32, 33, 34, 35, 36]. In particular, embedment was achieved by drop casting in succession 10 μL of a solution containing 100 μm protein and 20 mm phosphate buffer (pH 7.4), left at room temperature for 30 min, and 20 μL of a solution containing 10% m/m of gelatin type A (Sigma‐Aldrich, St Louis, Missouri, USA, G1890) [31, 33] and 20 mm phosphate buffer (pH 7.4), followed by incubation at room temperature for 5 min. The CV measurements were performed using 20 mm phosphate buffer at pH 7.4 as the working solution. The voltammetric signals are reproducible and persist for several cycles, indicating a stable protein layer. Currents increase linearly with increasing the scan rate (not shown), and their ratio is almost unit throughout the temperature range studied, as expected for an adsorbed protein. The formal potentials *E*°' were calculated from the average of the anodic and cathodic peak potentials. The electrocatalytic reduction of hydrogen peroxide by immobilized MB was studied in the O_2_‐free cell (kept under argon atmosphere) at 20 °C and pH 7.4 by gradually adding aliquots of H_2_O_2_ solutions to the working solutions. CVs were imported in MATLAB for background subtraction, after which peak current was read. The catalytic currents were measured as the difference between the cathodic peak current in the presence (*i_obs_*) and in the absence of a given H_2_O_2_ concentration (*i_0_*). Normalized catalytic currents at various H_2_O_2_ concentrations were calculated as the ratio between the catalytic currents (*i_obs_‐i_0_*) and cathodic peak current in the absence of H_2_O_2_ (*i_0_*).

## Conflict of interest

The authors declare no conflict of interest.

## Author contributions

SH planned experiments, performed experiments, analyzed data, and wrote the paper. MP performed experiments and analyzed data. MB planned experiments, analyzed data, and wrote the paper. CAB planned experiments and wrote the paper. GDR performed experiments and analyzed data. GR wrote the paper. PGF planned experiments and wrote the paper. CO wrote the paper. MS wrote the paper. GB planned experiments, analyzed data, and wrote the paper.

## Supporting information


**Fig S1**. (A) Experimental (solid lines) and simulated (dashed lines) low‐temperature (10K) cw‐EPR spectra of 50 µm wt MB (black) and H98Y MB variant (red) in 1 × PBS buffer, pH 7.4. (B) Electronic circular dichroism spectra in the far‐UV region of 5 µm wt MB (black) and H98Y MB variant (red) in 5 mm phosphate buffer, pH 7.5.
**Fig S2**. (A) Temperature induced unfolding of 5 µm wt MB (black) and 5 µm H98Y MB variant, followed by CD spectroscopy at 222 nm in 5 mm phosphate buffer, pH 7.5. (B) Thermogram obtained by differential scanning calorimetry of 10 µm wt MB (black) and H98Y MB (red). (C) *T*_m_ values of both proteins (wt MB, black circles; H98Y MB, red circles) plotted against the measured pH value.
**Fig S3**. (A) Representative spectral transitions from ferric wt MB and H98Y (black line) to the cyanide bound low‐spin heme protein (red line) after 60 s. (B) Binding constants *k*_on_, *k*_off_, and *K*_D_ were derived from plotting *k*_obs_ values from wt MB (gray circles) and H98Y MB (white squares) versus the cyanide concentration.
**Fig S4**. Kinetics constants for the reaction with H_2_O_2_ at different pH.
**Fig S5**. Cyclic voltammograms for wt MB (A) and the H98Y variant (B) immobilized onto a polycrystalline graphite electrode through embedment into hydrogel made of type A gelatin at increasing micromolar concentrations of H_2_O_2_.
**Fig S6**. Lineweaver‐Burk plots for the electrocatalytic reduction of H_2_O_2_ by wt MB (black) and H98Y MB (red) adsorbed onto a polycrystalline graphite electrode through embedment into a hydrogel made of type A gelatin at *T* = 293 K and pH 7.4.
