# Supplementary material for: Pseudoperoxidase activity, conformational stability, and aggregation propensity of the His98Tyr myoglobin variant: implications for the onset of myoglobinopathy
Source: FEBS J. 2021 Nov 3;289(4):1105–17. doi: 10.1111/febs.16235 (PMC9298411; doi:10.1111/febs.16235)
Supplement: Supplementary file 1 — Fig S1. (A) Experimental (solid lines) and simulated (dashed lines) low‐temperature (10K) cw‐EPR spectra of 50 µm wt MB (black) and H98Y MB variant (red) in 1 × PBS buffer, pH 7.4. (B) Electronic circular dichroism spectra in the far‐UV region of 5 µm wt MB (black) and H98Y MB variant (red) in 5 mm phosphate buffer, pH 7.5. Fig S2. (A) Temperature induced unfolding of 5 µm wt MB (black) and 5 µm H98Y MB variant, followed by CD spectroscopy at 222 nm in 5 mm phosphate buffer, pH 7.5. (B) Thermogram obtained by differential scanning calorimetry of 10 µm wt MB (black) and H98Y MB (red). (C) T m values of both proteins (wt MB, black circles; H98Y MB, red circles) plotted against the measured pH value. Fig S3. (A) Representative spectral transitions from ferric wt MB and H98Y (black line) to the cyanide bound low‐spin heme protein (red line) after 60 s. (B) Binding constants k on, k off, and K D were derived from plotting k obs values from wt MB (gray circles) and H98Y MB (white squares) versus the cyanide concentration. Fig S4. Kinetics constants for the reaction with H2O2 at different pH. Fig S5. Cyclic voltammograms for wt MB (A) and the H98Y variant (B) immobilized onto a polycrystalline graphite electrode through embedment into hydrogel made of type A gelatin at increasing micromolar concentrations of H2O2. Fig S6. Lineweaver‐Burk plots for the electrocatalytic reduction of H2O2 by wt MB (black) and H98Y MB (red) adsorbed onto a polycrystalline graphite electrode through embedment into a hydrogel made of type A gelatin at T = 293 K and pH 7.4. [file FEBS-289-1105-s001.zip › febs16235-sup-0001-FigS1-S6.pdf]

FEBS PRESS science publishing by scientists

WILEY

The FEBS Journal

www.febsjournal.org

Pseudoperoxidase activity, conformational stability, and aggregation propensity of the His98Tyr myoglobin variant: implications for the onset of myoglobinopathy

Stefan Hofbauer, Marcello Pignataro, Marco Borsari, Carlo Augusto Bortolotti, Giulia Di Rocco, Gianina Ravenscroft, Paul G. Furtmüller, Christian Obinger, Marco Sola and Gianantonio Battistuzzi

DOI: 10.1111/febs.16235

Supplementary information

Pseudoperoxidase activity, conformational stability and aggregation propensity of the His98Tyr myoglobin variant: Implications for the onset of myoglobinopathy.

Stefan Hofbauer[1], Marcello Pignataro[2], Marco Borsari[2], Carlo Augusto Bortolotti[3], Giulia Di Rocco[3], Gianina Ravenscroft[4,5], Paul G. Furtmüller[1], Christian Obinger[1], Marco Sola[3] and Gianantonio Battistuzzi[2,*]

[1] *Institute of Biochemistry, Department of Chemistry, University of Natural Resources and Life Sciences, Muthgasse 18, A-1190, Vienna, Austria*

[2] *Department of Chemical and Geological Sciences, University of Modena and Reggio Emilia, via Campi 103, 41125 Modena, Italy*

[3] *Department of Life Sciences, University of Modena and Reggio Emilia, via Campi 103, 41125 Modena, Italy*

[4] *Harry Perkins Institute of Medical Research, Nedlands, WA, Australia*

[5] *School of Biomedical Sciences, University of Western Australia, Nedlands, WA, Australia*

*****corresponding author: Prof. Gianantonio Battistuzzi
Department of Chemical and Geological Sciences,
University of Modena and Reggio Emilia
via Campi n 103, 41126 Modena, Italy
tel: 059-2058639
E-mail: gianantonio.battistuzzi@unimore.it
Orcid: 0000-0003-4716-5745

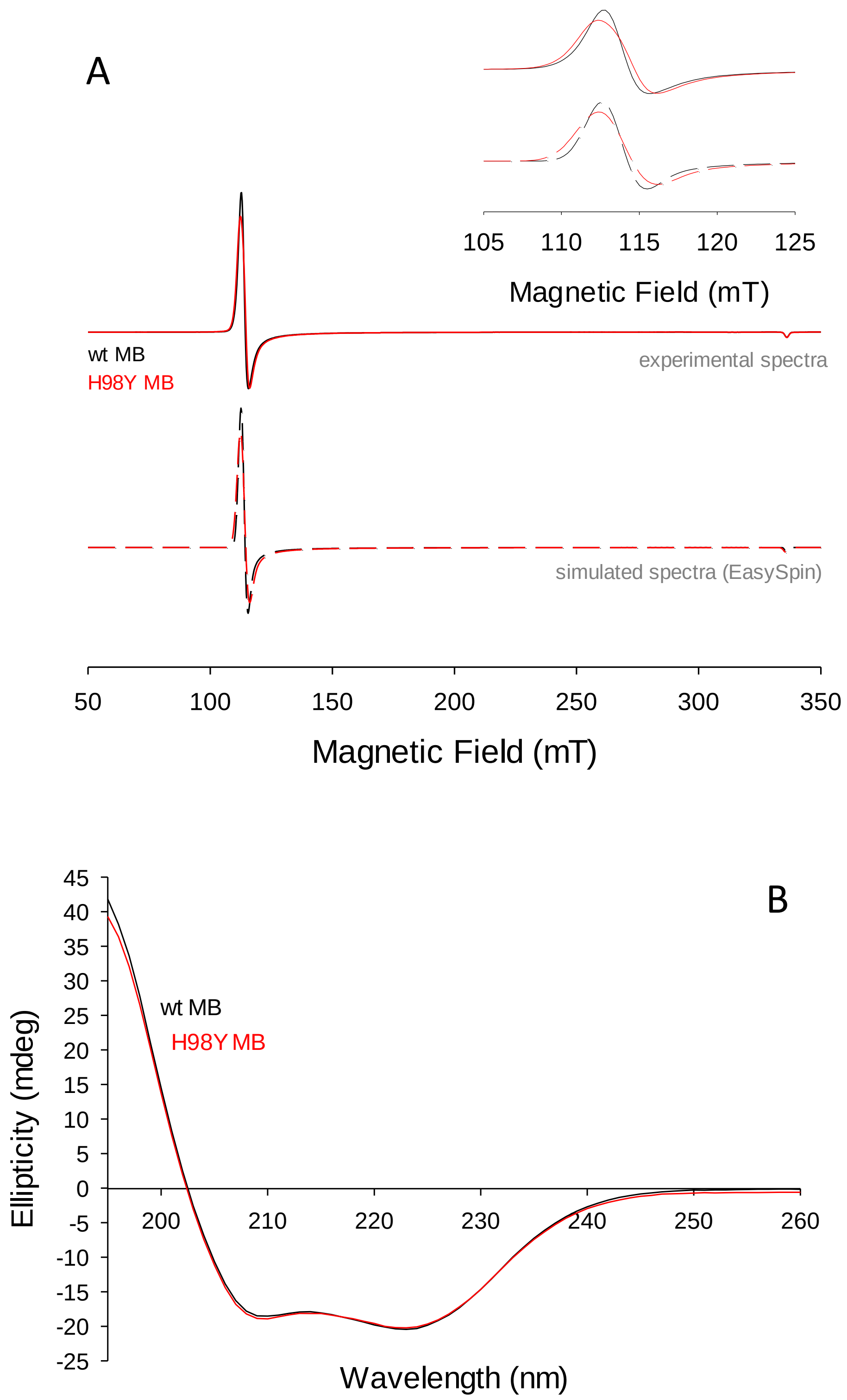

Figure S1. (A) Low-temperature (10K) cw-EPR spectra of 50 µM wt MB (solid black line) and H98Y MB variant (solid red line) in 1 × PBS buffer, pH 7.4. Spectra were simulated using the EasySpin software and are depicted as black dashed line (wt MB) and red dashed line (H98Y MB). The inset depicts an enlargement of the g_x/g_y region. **(B)** Electronic circular dichroism spectra in the far-UV region of 5 µM wt MB (black) and H98Y MB variant (red) in 5 mM phosphate buffer, pH 7.5.

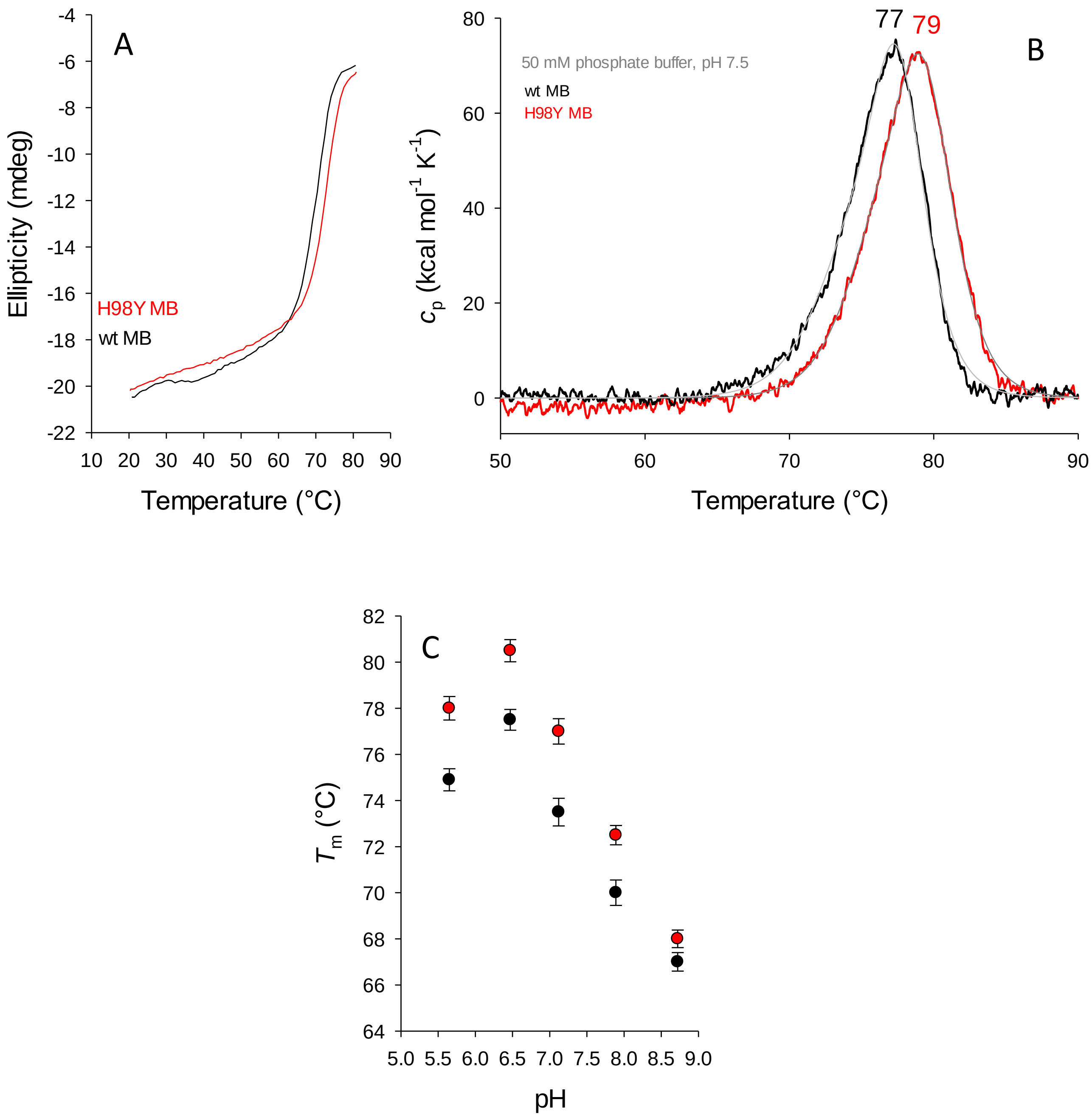

Figure S2. (A) Temperature induced unfolding of 5 µM wt MB (black) and 5 µM H98Y MB variant, followed by CD spectroscopy at 222 nm in 5 mM phosphate buffer, pH 7.5. (B) Thermogram obtained by differential scanning calorimetry of 10 µM wt MB (black) and H98Y MB (red). Least-square fits are depicted as grey lines. Buffer condition: 50 mM phosphate buffer, pH 7.5. (C) T_m values of both proteins (wt MB, black circles; H98Y MB, red circles) plotted against the measured pH value. The error bars represent the uncertainty of the sigmoidal fits, which were used to calculate the transition points from the thermo-CD experiments.

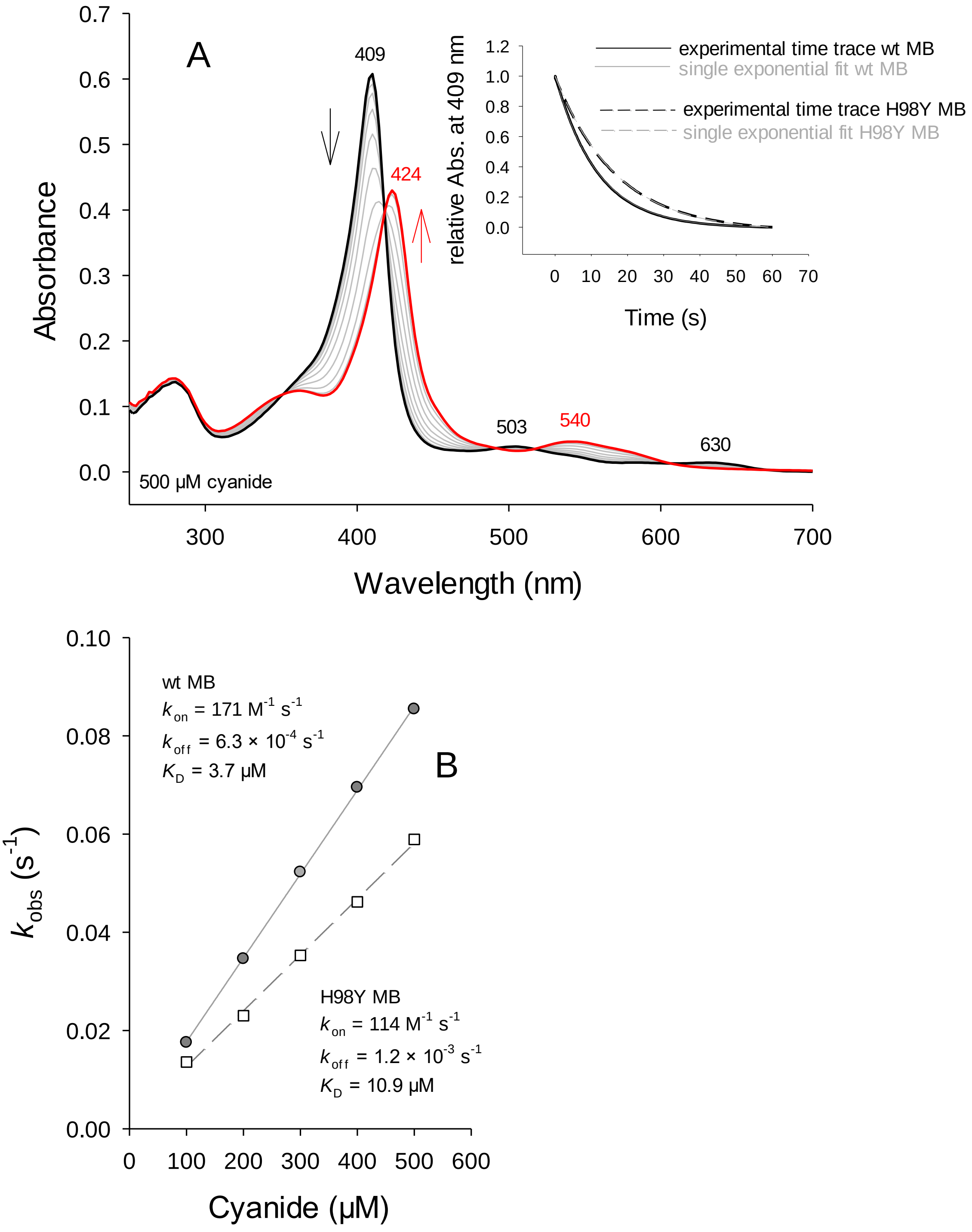

Figure S3. A) Representative spectral transitions from ferric wt MB and H98Y (black line) to the cyanide bound low-spin heme protein (red line) after 60 s. Grey lines depict the time-dependent intermediate spectral species. The inset depicts time traces (black lines) and single exponential fits (grey lines) upon addition of 500 µM cyanide to 5 µM wt Mb (solid lines) and H98Y (dashed lines). B) Binding constants k_{on}, k_{off}, and K_D were derived from plotting k_{obs} -values from wt MB (grey circles) and H98Y MB (white squares) *versus* the cyanide concentration. k_{on} was derived from the slope of the plots and k_{off} from the respective intercepts.

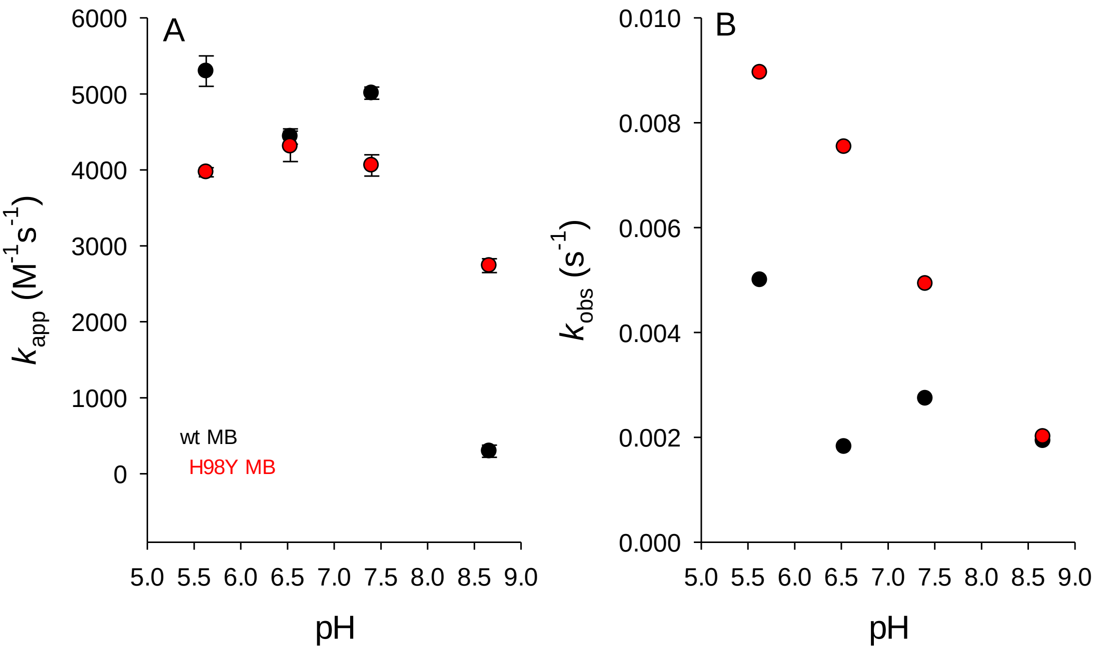

Figure S4. Kinetics constants for the reaction with H_2O_2 at different pH. a) k_{app} for the formation of Compound I, obtained by measuring k_{obs} at different H_2O_2 concentrations (see Methods). The error bars show the standard deviation (SD) of the triplicate (n = 3) measurements. B) k_{obs} for the heme bleaching reaction (second phase of double exponential fits), measured only with 3 µM wt MB and 3 µM H98Y MB and 5 mM H_2O_2 at pH 7.4

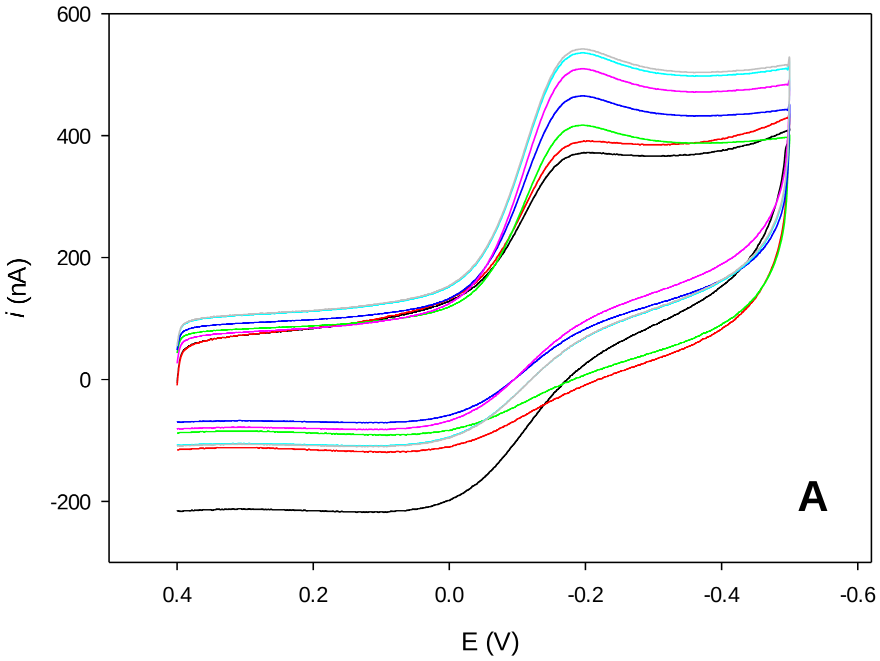

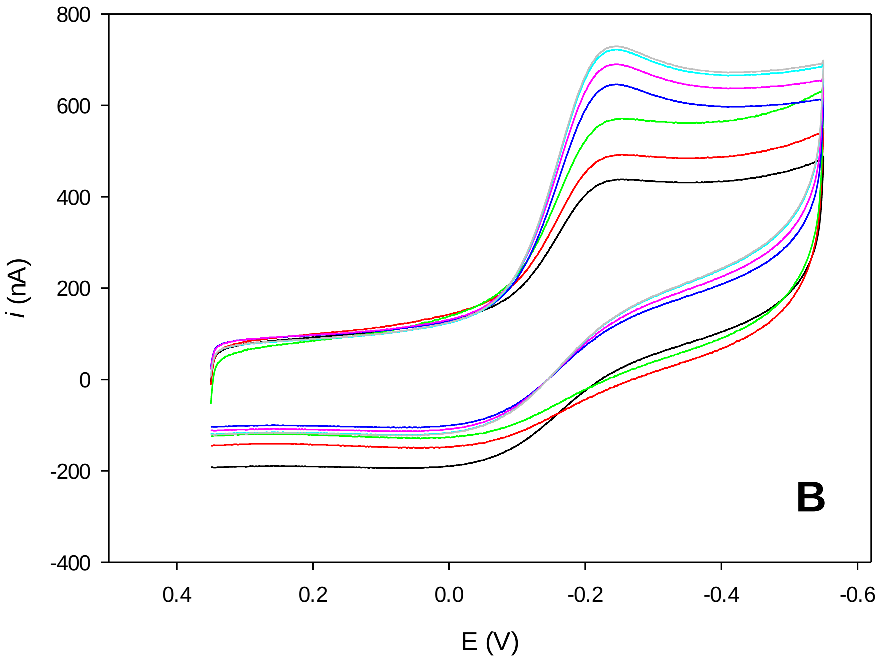

Figure S5. Cyclic voltammograms for wt MB (A) and the H98Y variant (B) immobilized onto a polycrystalline graphite electrode through embedment into hydrogel made of type-A gelatin at increasing micromolar concentrations of H_2O_2. (black, 0 μM; red, 10 μM; green, 20 μM; blue, 30 μM; magenta, 40 μM; cyan, 50 μM; gray, 60 μM). Working solution: 20 mM phosphate buffer, pH 7.4, Sweep rate, 50 mV s^{-1}. T = 293 K.

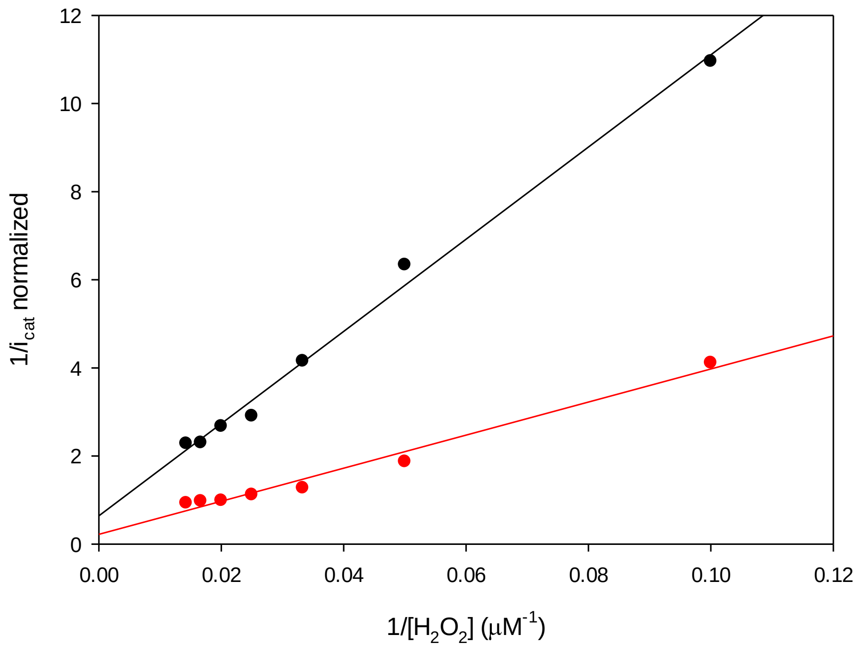

Figure S6. Lineweaver-Burk plots for the electrocatalytic reduction of H_2O_2 by wt MB (black) and H98Y MB (red) adsorbed onto a polycrystalline graphite electrode through embedment into a hydrogel made of type-A gelatin at T = 293 K and pH 7.4.
